# Increased Plasma Level of Longevity Protein Klotho as a Potential Indicator of Cognitive Function Preservation in Patients With Schizophrenia

**DOI:** 10.3389/fnins.2020.00610

**Published:** 2020-06-16

**Authors:** Jian-wen Xiong, Jin-qiong Zhan, Tao Luo, Hai-bo Chen, Qi-gen Wan, Yan Wang, Bo Wei, Yuan-jian Yang

**Affiliations:** ^1^Department of Psychiatry, Jiangxi Mental Hospital/Affiliated Mental Hospital of Nanchang University, Nanchang, China; ^2^Biological Psychiatry Laboratory, Jiangxi Mental Hospital/Affiliated Mental Hospital of Nanchang University, Nanchang, China

**Keywords:** schizophrenia, cognitive function, klotho, plasma, correlation

## Abstract

Cognitive impairments are a core feature of schizophrenia. Klotho is an anti-aging protein with demonstrated cognitive-enhancing effects on the brain. The purpose of this study was to investigate the differences in levels of plasma klotho between patients with schizophrenia and healthy controls, as well as the relationship between klotho level and cognitive function in patients. Forty patients with schizophrenia and 40 gender- and age-matched healthy individuals were recruited. Positive and Negative Syndrome Scale (PANSS) was used to assess the psychopathology of patients. A neuropsychological battery was performed to evaluate the cognitive function of participants. Plasma klotho was measured using enzyme-linked immunosorbent assay. We show that patients with schizophrenia performed worse in the neurocognitive tests than the healthy controls. The levels of plasma klotho were significantly higher in schizophrenia patients than in healthy controls (*p* < 0.001). In patients, plasma klotho levels were positively correlated with cognitive function with regard to attention (*p* = 0.010), working memory (*p* < 0.001), verbal memory (*p* = 0.044), executive function (*p* < 0.001), and composite cognitive score (*p* < 0.001). Stepwise linear regression analysis shows that executive function had the highest correlation with plasma klotho levels (β = 0.896, *t* = 8.290, *p* < 0.001). Collectively, these results indicate that anti-aging protein klotho may be implicated in the pathogenesis of schizophrenia, and increased klotho may act as a compensatory factor for the preservation of cognitive function in schizophrenia. Further studies are needed to investigate the dynamic changes of klotho and the mechanisms by which klotho modulates cognition in schizophrenia.

## Introduction

Schizophrenia is a chronic severe mental disorder affecting approximately 1% of the world’s population. Besides the positive and negative symptoms, cognitive impairments are also considered as a core feature of schizophrenia and have a negative impact on occupational, social, and economic functions of patients (Medalia and Saperstein, 2013; Owen et al., 2016; Lystad et al., 2017). Several domains of cognition have been shown to be impaired in patients with schizophrenia, including the speed of information processing, verbal learning, working memory, attention, and executive function (Wu et al., 2016; Xiong et al., 2018; Zhang et al., 2019). Although many studies have been conducted to try to elucidate the underlying molecular mechanisms, the related biological pathways that are disrupted to result in cognitive impairments are still not clear.

Klotho is a transmembrane protein that was firstly linked to longevity (Kurosu et al., 2005). Mice lacking the KLOTHO gene begin to display numerous phenotypes that are similar to normal aging of human being at the age of 3–4 weeks (Kuro-O et al., 1997). Recent studies show that klotho plays an important regulatory role in cognition (Abraham et al., 2016). Genetic reduction of klotho during embryogenesis in mice results in hypomyelination, synaptic alteration, and memory retention deficits (Nagai et al., 2003; Shiozaki et al., 2008; Chen et al., 2013). Overexpression of klotho promotes hippocampal synaptic plasticity and enhances learning and memory ability in normal mice (Dubal et al., 2014). Elevation of brain klotho also could reverse synaptic and cognitive impairments in the mouse model of Alzheimer’s disease (Dubal et al., 2015; Zeng et al., 2019). The underlying mechanisms of elevated klotho on cognition involve the optimization of synaptic N-methyl-D-aspartate receptor (NMDAR) function in the hippocampus and frontal cortex (Dubal et al., 2014, 2015), in which the hypofunction of NMDARs was found in schizophrenia. Klotho is widely expressed in a variety of human tissues, particularly in the kidney, the parathyroid gland, and the choroid plexus (Imura et al., 2004). In the brain, klotho is produced primarily by the choroid plexus and then secreted into the cerebrospinal fluid (CSF) (Imura et al., 2004; German et al., 2012). It circulates throughout the brain and body following cleavage by ADAM 10 and 17 from its transmembrane form (Chen et al., 2007). The pleiotropic effect of klotho in mice has led to several studies on the relationship of klotho and cognitive function in humans. Luo et al. (2015) reported that individuals with mild cognitive impairment have much higher methylation levels of klotho promoter. One study conducted in aging Caucasian individuals without dementia or cognitive complaints revealed that a haplotype and functional variant of the KLOTHO gene, KL-VS, was common in these populations, and the KL-VS allele was associated with increased serum klotho levels and better cognitive performance (Dubal et al., 2014). Similarly, Deary et al. (2005) showed that people with the KLOTHO V/V genotype had lower cognitive ability than those with the heterozygotes.

Two studies have been conducted to investigate the linkage of klotho with schizophrenia. A genetic study reported an adverse effect of KL-VS heterozygosity on immediate memory in patients with schizophrenia, which is opposite to that seen in healthy individuals (Morar et al., 2018). Another study showed that patients with schizophrenia had higher levels of serum klotho compared with controls, although no significant difference was found between two groups (Yazici et al., 2019). However, whether plasma klotho is associated with the neurocognitive functions in schizophrenia patients is still unclear. To fill this research gap, this article investigated the difference in plasma klotho levels between patients with schizophrenia and healthy controls. Additionally, the correlation between plasma klotho levels and cognitive function in schizophrenia patients was examined. In the view of the regulatory role of klotho in cognition and NMDAR function, we hypothesized that altered klotho was associated with the cognitive deficits in schizophrenia.

## Materials and Methods

### Subjects

A total of 40 schizophrenia inpatients were recruited from Jiangxi Mental Hospital. These patients have stopped their medicines at their own decision, had acute exacerbation for their psychotic symptoms, and were admitted into acute ward for assessment and treatment. Patients were selected for this study if they (1) were between 18 and 60 years old; (2) had at least 9 years of formal education; (3) were assessed with the Structured Clinical Interview for DSM-IV Axis I Disorders (SCID) by two psychiatrists and were diagnosed with schizophrenia; (4) have a total Positive and Negative Syndrome Scale (PANSS) score between 60 and 120 (inclusive); and (5) were not diagnosed with any other DSM-IV axis I or axis II disorders, current pregnancy, and other physical diseases including cerebral infarction over the past 3 months. Patients with neurological disorders, mental retardation, and drug/alcohol abuse or addiction were excluded from this study. Furthermore, to rule out the potential influence of antipsychotic medications on plasma klotho and cognitive function as much as possible, all these recruited subjects had not taken any antipsychotic drug for more than 3 months at the time of cognitive assessment and klotho measurement.

Forty healthy individuals, who were matched with the patients by age, gender, education, and body mass index (BMI), were recruited from the local community as controls. Control individuals with a personal or family history of drug abuse or psychiatric disorders were excluded from the control group.

Given that some diseases including chronic inflammatory disease (Martin-Nunez et al., 2020), cardiovascular diseases (Keles et al., 2016; Ding et al., 2019), metabolic syndrome (Kim et al., 2019), hyperlipidemia (Sastre et al., 2013), diabetes (Flotynska et al., 2018),acute and chronic kidney injury (Doi et al., 2011), neoplasms (Brominska et al., 2019), and liver damage (Dongiovanni et al., 2020) may lead to changes in plasma klotho, both patient and control groups were assessed with a medical history, complete physical examination, electrocardiogram, laboratory tests, and subjects with abnormal parameters for the assessment of diseases described above were excluded in this study.

The research was conducted in accordance with the Declaration of Helsinki, and all procedures for this study were approved by the Institutional Review Board at Jiangxi Mental Hospital. A written informed consent was obtained from each subject or his or her legal guardians before start of this study.

### Measurements of Psychopathology and Cognition

We adopted the Positive and Negative Syndrome Scale (PANSS) to evaluate the severity of psychopathological symptoms of patients. In order to insure the consistency and reliability of rating across the evaluations, a training of how to use the PANSS was carried out among the psychiatrists who participated in this study. For PANSS total score, the inter-observer correlation coefficient was higher than 0.80 after the training.

The cognitive functions of subjects were assessed with a comprehensive battery of neurocognitive tests, which are widely used in China, and their validity and clinical reliability have been well proved in Chinese populations (Wu et al., 2016; Xiong et al., 2018). The battery consists of the following seven tests:

The trail making test part A (TMT-A): In this test, the participant is asked to draw a line to link consecutively the numbered circles that are placed irregularly on a sheet of paper. They need to draw as fast as possible and maintain accuracy. The measured outcome is the time required to accomplish this test.

Brief assessment of cognition in schizophrenia (BACS) symbol coding: This test contains 133 digit-symbol pairs. The subject needs to sign the corresponding symbol for a given number as fast as possible. The measured outcome is the number of correct symbols listed during 120 s.

The Wechsler Memory Scale-3rd edition spatial span (WMS-III spatial span): We irregularly space 10 cubes on a board. The subject is required to remember the order that we point to a string of cubes in both the forward and reverse direction. At each level, different combinations are given for twice trials. The measured outcome is the number of correctly recalled trials in each situation.

The brief visual-spatial memory test-revised (BVMT-R): This task contains reproducing six geometric figures from memory. The administrator displays these figures three times with each time for 10 s. The subject needs to draw as many figures as possible in the correct locations on a page in the response booklet. The measured outcome is the total number of correctly recalled figures.

The Hopkins verbal learning test-revised (HVLT-R): A list of 12 words from three taxonomic categories is presented in this test. The subject is asked to recall as many words as possible. This task consists of three trials of learning and a trial of delayed recall (25–30 min delay). The total number of correctly recalled words is recorded as the measured outcome.

The Stroop color-word test (SCWT): Three parts are included in this task: word page (the colors’ names are printed in black ink), color page (rows of Xs printed in colored ink), and word-color page (the words from the first page are printed in the colors from the second page, while the meaning of word is not match with the ink color). There are 100 items in each trial. The subject is required to read as quickly as possible within 45 s. The measured outcome is the number of correct names in each trial.

Continuous performance test-identical pairs (CPT-IP): In this test, numbers with 2, 3, and 4 digits are flashed briefly in a computer monitor, and the subject is required to click the mouse when the same number shows consecutively. The total hits number is <90, the total false alarms number is also <90, and the total random responses number is <270.

### Plasma Klotho Measurement

Whole blood was obtained from the forearm vein and collected into a tube with EDTA between 7:00 and 9:00 a.m. following an overnight fasting. The plasma was separated, aliquoted, and stored at temperature of −80°C before klotho measurement. The levels of plasma klotho were determined using sandwich enzyme-linked immunosorbent assay (ELISA) kits that are available on market (Wuhan USCN Business, Wuhan, China). The sensitivity for klotho was 6.2 pg/ml, and the inter- and intra-assay variation coefficients were lower than 10 and 12%, respectively. The absorbencies were examined at 450 nm using a microtiter analyzer. We used the same assay batches to assay the samples of the subjects. Each sample was assayed in duplicate.

### Statistical Analysis

Data were analyzed with the Statistical Product and Service Solutions (SPSS) 18.0 software. Variables are presented as mean ± standard deviation (SD). We used the chi-squared test to compare categorical variables between patients with schizophrenia and healthy controls. The Student’s *t*-test was performed to compare the continuous variables that were distributed normally, and the Kolmogorov–Smirnov and Mann-Whitney *U* tests were conducted to compare the independent variables that did not fit the normal distribution. Linear regression analysis was used to assess the potential relationships between the plasma klotho levels and the psychopathological symptoms or cognitive function in patients. Multivariate stepwise linear regression analysis was performed to determine the most parsimonious model for correlations with klotho, controlling for age, sex, education, and BMI. Standardized coefficients (*B* values) with 95% confidence intervals (95% CI) were calculated. All statistical tests were two-tailed, and the level of significance was established as *p* < 0.05.

## Results

A total of 40 patients with schizophrenia (22 males and 18 females; age: 30.36 ± 7.97 years) and 40 healthy controls (19 males and 21 females; age: 33.23 ± 8.57 years) were recruited in this study (Table 1). The mean education years of controls and patients were 12.13 ± 4.83 and 11.95 ± 3.27 years, respectively, (*p* > 0.05). From the schizophrenia patients, the age of onset was 26.98 ± 6.73 years; the illness duration was 6.52 ± 5.17 years, and the times of psychiatric admission was 2.63 ± 1.44. The body mass index (BMI) in patient group was higher than that in healthy controls (*p* = 0.044). In addition, patients with schizophrenia performed worse in nearly all of the neurocognitive tests than the healthy controls (all *p* < 0.05) except for the BVMT-R (*p* = 0.063).

**TABLE 1:** Comparisons of demographic data, psychopathology and cognition between healthy controls and patients with schizophrenia.

	Healthy controls (*n* = 40)	Patients (*n* = 40)	Statistic value	*p*-value
**Demographic data**				
Age (years)	33.23 ± 8.57	30.36 ± 7.97	–1.449	0.147
Gender (M/F)	19/21	22/18	0.450	0.502
Education (years)	12.13 ± 4.83	11.95 ± 3.27	–0.015	0.988
BMI (kg/m^2^)	20.94 ± 1.82	21.82 ± 2.05	–2.044	0.044*
Age of onset (years)	N/A	26.98 ± 6.73	N/A	N/A
Duration of illness	N/A	6.52 ± 5.17	N/A	N/A
**Psychopathology**				
Positive symptoms score	N/A	21.75 ± 4.15	N/A	N/A
Negative symptom score	N/A	14.15 ± 4.28	N/A	N/A
General symptoms score	N/A	44.35 ± 5.49	N/A	N/A
Total score of PANSS	N/A	81.03 ± 10.33	N/A	N/A
**Cognitive function**				
TMT-A	35.58 ± 8.25	75.42 ± 26.30	–6.961	< 0.001***
BACS-SC	65.38 ± 6.00	34.16 ± 13.56	–7.293	< 0.001***
CPT-IP	3.39 ± 0.93	1.48 ± 0.77	–6.678	< 0.001***
WMS-III-SS	16.93 ± 2.67	14.68 ± 3.25	–2.295	0.022*
HVLT-R	26.65 ± 4.38	19.33 ± 5.78	6.384	< 0.001***
BVMT-R	27.10 ± 9.98	24.00 ± 7.73	–1.926	0.063
Stroop word score	85.90 ± 8.52	55.15 ± 14.09	11.810	< 0.001***
Stroop color score	51.28 ± 8.89	35.29 ± 14.25	6.020	< 0.001***
Stroop color-word score	36.42 ± 7.89	21.22 ± 11.48	–5.701	< 0.001***

Data are presented as mean ± SD; BMI, body mass index; PANSS, Positive and Negative Syndrome Scale; TMT-A, trail making task part A; BACS-SC, brief assessment of cognition in schizophrenia-symbol coding; CPT-IP, continuous performance test-identical pairs; WMS-III-SS, the Wechsler Memory Scale-3rd edition-spatial span; HVLT-R, the Hopkins verbal learning test-revised; BVMT-R, brief visual-spatial memory test-revised. *p < 0.05; ***p < 0.001.

**FIGURE 1:**
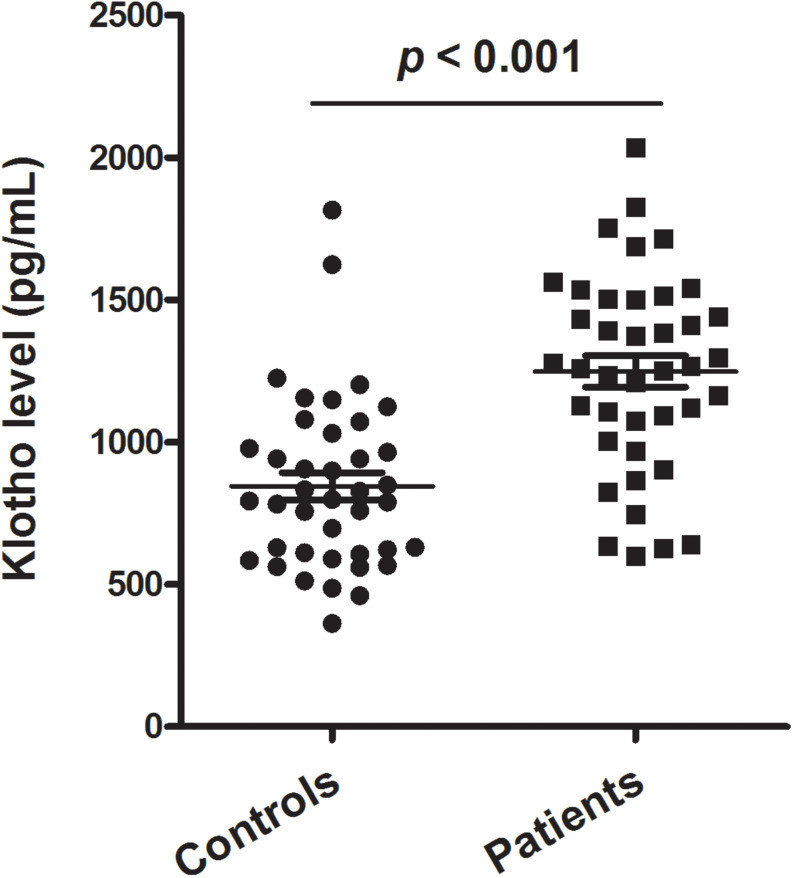
Plasma levels of klotho in healthy controls (*n* = 40) and patients with schizophrenia (*n* = 40). The sample means are indicated by the black bars.

Figure 1 shows the levels of plasma klotho in control and patient groups. The Kolmogorov–Smirnov test showed that plasma klotho levels were distributed normally in both the patient (*p* = 0.200) and the control group (*p* = 0.200), and thus, Student’s *t*-test was used to compare klotho levels between two groups. We found that patients with schizophrenia had significantly higher levels of plasma klotho than did healthy controls (controls: 844.49 ± 302.94 pg/mL; patients: 1233.25 ± 382.25 pg/mL; *t* = −5.041, *p* < 0.001).

Table 2 shows the correlations between plasma klotho levels and demographic data, psychopathology and cognitive function in patients with schizophrenia. We grouped the seven cognitive tests into six domains: processing speed (TMT-A, BACS-symbol coding), attention (CPT-IP), working memory (WMS-III spatial span), verbal learning (HVLT-R), visual memory (BVMT-R), and executive function (Stroop color-word test). To calculate the cognitive score of each domain, the primary measure of each test was first converted to standardized *z*-score by setting the mean of each measure to zero and the standard deviation to one. For domain with more than one test, cognitive domain score was determined by calculating the mean of the *z*-scores for the measures that comprised the domain, then converting the mean to a *z*-score. Finally, a composite score was calculated by averaging all of the six domain *z*-scores from the neurocognitive tests and then calculating a *z*-score of the composite (Keefe et al., 2004). The *z*-scores for each domain were then used for analysis.

**TABLE 2:** Correlations between plasma klotho levels, demographic variables, psychopathology and cognitive function in patients with schizophrenia (*n* = 40).

Variables	Klotho		
	*B* (95% CI)	*t*	*p*-value
**Demographic data**			
Age (years)	0.023(−0.332,0.377)	0.132	0.896
Gender (M/F)	0.165(−0.129,0.458)	1.165	0.257
Education (years)	−0.060(−0.315,0.195)	–0.488	0.631
BMI (kg/m^2^)	−0.116(−0.354,0.122)	–1.008	0.324
Age of onset (years)	0.251(0.062,0.440)	2.692	0.011*
Duration of illness (years)	0.078(−0.324,0.480)	0.403	0.691
**Psychopathology**			
Positive symptoms score	−0.124(−0.509,0.261)	–0.670	0.510
Negative symptoms score	−0.136(−0.655,0.384)	–0.541	0.594
General symptoms score	−0.427(−0.992,0.145)	–1.548	0.136
Total score of PANSS	0.666(0.272,1.604)	1.472	0.155
**Cognitive function**			
Processing speed (TMT-A, BACS)	−0.243(−0.676,0.189)	–1.166	0.256
Attention (CPT-IP)	0.303(0.078,0.528)	2.733	0.010*
Working memory (WMS-III-SS)	0.414(0.207,0.621)	4.052	< 0.001***
Verbal learning (HVLT-R)	0.324(0.009,0.639)	2.127	0.044*
Visual memory (BVMT-R)	−0.147(−0.672,0.377)	–0.583	0.566
Executive function (SCWT)	0.896(0.677,1.116)	8.290	< 0.001***
Composite cognitive score	0.422(0.217,0.627)	4.171	< 0.001***

*p < 0.05; ***p < 0.001.

For patients, linear regression analysis showed that plasma levels of klotho were positively correlated with age of onset (*p* = 0.011) and cognitive function with regard to attention (*p* = 0.010), working memory (*p* < 0.001), verbal memory (*p* = 0.044), executive function (*p* < 0.001), and composite cognitive score (*p* < 0.001). The levels of klotho had no significant correlation with psychopathological symptoms (all *p* > 0.05). In this model of regression analysis, the following variables were included: age of onset (years), attention (CPT-IP), working memory (WMS-III-SS), verbal learning (HVLT-R), executive function (SCWT) and composite cognitive score, and the adjusted *R*^2^ was 0.690. Finally, we conducted the analysis with multivariate stepwise linear regression by controlling for age, sex, education, and BMI to determine the most parsimonious model for correlations with klotho (adjust *R*^2^ = 0.970) and found that executive function had the highest correlation with plasma klotho levels (β = 0.896, *t* = 8.290, *p* < 0.001).

## Discussion

This study demonstrates that patients with schizophrenia had higher plasma levels of klotho in relation to healthy controls, and plasma klotho was positively correlated with cognitive function in patients with schizophrenia. To our knowledge, this is the first study to report the correlation between plasma klotho and cognitive function in schizophrenia.

Klotho is a lifespan-associated protein that displays cognitive-enhancing effect (Abraham et al., 2016). KL-VS is a single allele of the KLOTHO gene. Normally, the heterozygote KL-VS gene could result in an increase in circulating klotho levels (Di Bona et al., 2014). Higher klotho levels are associated with longer lifespans and better cognitive functions, while lower klotho levels are shown to be correlated with shorter lifespans and deteriorating cognition (Arking et al., 2002; Deary et al., 2005; Di Bona et al., 2014; Dubal et al., 2014). Morar et al. (2018) have performed a genetic study in patients with schizophrenia who had been classified into cognitively spared (CS) and cognitively deficient (CD) clusters and showed that heterozygous KL-VS carriers in the two subgroups displayed worse performances than non-carriers. Yazici et al. (2019) have measured the levels of serum Klotho in schizophrenia patients and found that serum klotho concentrations were increased in patients. Although the difference in serum levels of klotho between the patient and control group in their study did not show a statistical significance, they observed the highest values in the patients with acute exacerbation for psychotic symptoms and the lowest values in the healthy controls (Yazici et al., 2019). In this study, we found that patients with schizophrenia had significantly higher levels of plasma klotho than did healthy controls. Given that the recruited patients in this study had not received any antipsychotic drug for more than 3 months before taking part in this research, we presume that increased plasma klotho in schizophrenia patients is attributed to the schizophrenia *per se*, rather than a phenomenon secondary to antipsychotic medication. However, this presumption still needs to be confirmed by detecting klotho levels in first-episode and drug-naive patients with schizophrenia.

Cognitive deficits are considered as a core feature of schizophrenia (Owen et al., 2016). Consistent with previous reports (McCleery et al., 2014; Wu et al., 2016; Xiong et al., 2018), this study showed that schizophrenia patients had worse performances in the tests of information processing speed, attention, working memory, verbal learning, and executive function relative to the healthy individuals. Several studies have demonstrated that klotho exerts a cognitive-enhancing effect by regulating the antioxidative defense and NMDAR function in the brain (Dubal et al., 2014, 2015; Zhou et al., 2018). Mice with *KLOTHO* mutation displayed impaired cognitive function and increased lipid and DNA peroxide levels in the hippocampus (Yamamoto et al., 2005; Shin et al., 2014). Treating these mice with a potent antioxidant, alpha-tocopherol, improved cognitive function and reduced the accumulation of lipid peroxide and the number of apoptotic cells (Nagai et al., 2003). Klotho elevation could promote synaptic plasticity and cognition by enriching GluN2B subunit of NMDARs at synapses in the hippocampus and frontal cortex in both normal and Alzheimer disease (AD) model mice (Dubal et al., 2014, 2015). Zhou et al. (2018) reported that klotho upregulation in the brain reversed aging-associated memory deficits and oxidative stress damage. Here, we found that plasma klotho levels were increased in patients with schizophrenia, and klotho levels were positively correlated with attention, working memory, verbal memory, and executive capacity in patients, indicating that klotho dysregulation might contribute to the cognitive impairments in schizophrenia.

It should be noted that we had hypothesized that the level of plasma klotho was decreased in patients with schizophrenia because klotho plays a beneficial role in cognition, and cognitions are impaired in schizophrenia. However, contrary to our expectation, the plasma levels of klotho were found to be increased in schizophrenia patients. Intriguingly, elevated plasma klotho was positively correlated with the domains of cognitive tests including attention, working memory, verbal memory, and executive capacity in patients with schizophrenia, which is similar to the relationship observed in healthy individuals (Dubal et al., 2014; Morar et al., 2018). These findings suggest an assumption that elevated klotho may serve as a compensatory factor for cognitive function preservation in schizophrenia. It is possible that the pathogenic cell environment in patients positively affects klotho activity, counteracting the cognitive impairments of patients with schizophrenia. Oxidative damage to the brain caused by excessive reactive oxygen species has been implicated in the psychopathology of cognitive impairments in schizophrenia (Wang et al., 2018; Xie et al., 2019). Klotho protein has an ability to protect organ against oxidative stress damage (Nagai et al., 2003; Ji et al., 2018; Zhou et al., 2018). Perhaps, accumulated reactive oxygen species might stimulate the body to produce more klotho protein to alleviate the oxidative damage. Furthermore, hypofunction of NMDARs in the brain of schizophrenia patients might also trigger a compensatory increase of klotho protein. However, these explanations are quite speculative. More studies are required to perform to elucidate the molecular mechanisms underlying plasma klotho elevation in schizophrenia.

There are some limitations in this study. First, we measured klotho levels in plasma, but not in the brain tissue or cerebral spinal fluid (CSF). Whether plasma klotho levels could reflect the concentrations of klotho protein in the central nervous system is unclear. Second, this was a cross-sectional study. Although increased klotho was correlated with cognitive deficits in schizophrenia patients, the causal relationship between klotho levels and cognitive impairments is still undetermined. Third, the sample size was relatively small. Replication in larger samples is needed to validate this conclusion. Fourth, although an elevated klotho level was found in patients with schizophrenia, the mechanisms underlying klotho elevation and the mechanisms by which klotho modulates cognition in schizophrenia are needed to be addressed. Finally, medication-free schizophrenia patients with acute exacerbation were enrolled in this study. These patients were clinically unstable (PANSS score between 60 and 120). Although previous studies have also demonstrated that schizophrenia patients who were clinically unstable could be successfully assessed for the cognitive function (Yu et al., 2018; Zhang et al., 2018), the unstable status of patients would weaken the reliability of cognitive assessment. Furthermore, the patient who participated in research should be clinically stable for ethical reason. Thus, the findings of this study are still needed to be replicated in clinically stable patients.

## Conclusion

This study reveals that plasma klotho levels were significantly increased in patients with schizophrenia when compared to gender- and age-matched healthy controls. The levels of plasma klotho were positively associated with cognitive performance in schizophrenia patients. These results indicate that anti-aging protein klotho may be implicated in the pathogenesis of schizophrenia, and increased klotho may act as a compensatory factor for the preservation of cognitive function in schizophrenia. The finding of this study helps to deepen our understanding of the molecular mechanisms underlying schizophrenia-associated cognitive impairments. Furthermore, given that cognitive deficits are a core feature of schizophrenia and the best predictor of long-term functional outcome for schizophrenia patients, the finding that compensatory increase of plasma klotho was correlated with cognitive preservation in patients indicates that plasma klotho could be used as a predictor of functional outcomes in patients with schizophrenia. However, this is a preliminary study, and further research using prospective study and animal experiments is needed to investigate the dynamic changes of klotho and the causal relationship between klotho and cognitive impairments in schizophrenia.

## Data Availability Statement

The datasets generated for this study are available on request to the corresponding author.

## Ethics Statement

The studies involving human participants were reviewed and approved by the Institutional Review Board at Jiangxi Mental Hospital. The patients/participants provided their written informed consent to participate in this study.

## Author Contributions

YY, JX, TL, HC, JZ, QW, and YW participated in clinical data collection and lab data analysis. YY and BW designed the study, analyzed the data, and prepared the manuscript. All authors read and approved the final manuscript.

## Conflict of Interest

The authors declare that the research was conducted in the absence of any commercial or financial relationships that could be construed as a potential conflict of interest.

## References

[B1] Abraham C. R. Mullen P. C. Tucker-Zhou T. Chen C. D. Zeldich E. (2016). Klotho is a neuroprotective and cognition-enhancing protein. *Vitam. Horm.* 101 215–238. 10.1016/bs.vh.2016.02.004 27125744

[B2] Arking D. E. Krebsova A. Macek M. Sr. Macek M. Jr. Arking A. Mian S. I. (2002). Association of human aging with a functional variant of klotho. *Proc. Natl. Acad. Sci. U.S.A.* 99 856–861. 10.1073/pnas.022484299 11792841 PMC117395

[B3] Brominska B. Gabryel P. Jarmolowska-Jurczyszyn D. Janicka-Jedynska M. Kluk A. Trojanowski M. (2019). Klotho expression and nodal involvement as predictive factors for large cell lung carcinoma. *Arch. Med. Sci.* 15 1010–1016. 10.5114/aoms.2018.75889 31360195 PMC6657266

[B4] Chen C. D. Podvin S. Gillespie E. Leeman S. E. Abraham C. R. (2007). Insulin stimulates the cleavage and release of the extracellular domain of Klotho by ADAM10 and ADAM17. *Proc. Natl. Acad. Sci. U.S.A.* 104 19796–19801. 10.1073/pnas.0709805104 18056631 PMC2148378

[B5] Chen C. D. Sloane J. A. Li H. Aytan N. Giannaris E. L. Zeldich E. (2013). The antiaging protein Klotho enhances oligodendrocyte maturation and myelination of the CNS. *J. Neurosci.* 33 1927–1939. 10.1523/jneurosci.2080-12.2013 23365232 PMC3711388

[B6] Deary I. J. Harris S. E. Fox H. C. Hayward C. Wright A. F. Starr J. M. (2005). KLOTHO genotype and cognitive ability in childhood and old age in the same individuals. *Neurosci. Lett.* 378 22–27. 10.1016/j.neulet.2004.12.005 15763166

[B7] Di Bona D. Accardi G. Virruso C. Candore G. Caruso C. (2014). Association of Klotho polymorphisms with healthy aging: a systematic review and meta-analysis. *Rejuvenation Res.* 17 212–216. 10.1089/rej.2013.1523 24164579

[B8] Ding J. Tang Q. Luo B. Zhang L. Lin L. Han L. (2019). Klotho inhibits angiotensin II-induced cardiac hypertrophy, fibrosis, and dysfunction in mice through suppression of transforming growth factor-beta1 signaling pathway. *Eur. J. Pharmacol.* 859:172549. 10.1016/j.ejphar.2019.172549 31325434

[B9] Doi S. Zou Y. Togao O. Pastor J. V. John G. B. Wang L. (2011). Klotho inhibits transforming growth factor-beta1 (TGF-beta1) signaling and suppresses renal fibrosis and cancer metastasis in mice. *J. Biol. Chem.* 286 8655–8665. 10.1074/jbc.m110.174037 21209102 PMC3048747

[B10] Dongiovanni P. Crudele A. Panera N. Romito I. Meroni M. De Stefanis C. (2020). beta-Klotho gene variation is associated with liver damage in children with NAFLD. *J. Hepatol.* 72 411–419. 10.1016/j.jhep.2019.10.011 31655133

[B11] Dubal D. B. Yokoyama J. S. Zhu L. Broestl L. Worden K. Wang D. (2014). Life extension factor klotho enhances cognition. *Cell Rep.* 7 1065–1076. 10.1016/j.celrep.2014.03.076 24813892 PMC4176932

[B12] Dubal D. B. Zhu L. Sanchez P. E. Worden K. Broestl L. Johnson E. (2015). Life extension factor klotho prevents mortality and enhances cognition in hAPP transgenic mice. *J. Neurosci.* 35 2358–2371. 10.1523/jneurosci.5791-12.2015 25673831 PMC4323521

[B13] Flotynska J. Uruska A. Araszkiewicz A. Zozulinska-Ziolkiewicz D. (2018). Klotho protein function among patients with type 1 diabetes. *Endokrynol. Pol.* 69 696–704. 10.5603/ep.a2018.0070 30620382

[B14] German D. C. Khobahy I. Pastor J. Kuro O. M. Liu X. (2012). Nuclear localization of Klotho in brain: an anti-aging protein. *Neurobiol. Aging* 33 e1425–e1430. 10.1016/j.neurobiolaging.2011.12.018 PMC3328593 22245317

[B15] Imura A. Iwano A. Tohyama O. Tsuji Y. Nozaki K. Hashimoto N. (2004). Secreted Klotho protein in sera and CSF: implication for post-translational cleavage in release of Klotho protein from cell membrane. *FEBS Lett.* 565 143–147. 10.1016/j.febslet.2004.03.090 15135068

[B16] Ji N. Luan J. Hu F. Zhao Y. Lv B. Wang W. (2018). Aerobic exercise-stimulated Klotho upregulation extends life span by attenuating the excess production of reactive oxygen species in the brain and kidney. *Exp. Ther. Med.* 16 3511–3517. 30233703 10.3892/etm.2018.6597 PMC6143843

[B17] Keefe R. S. Goldberg T. E. Harvey P. D. Gold J. M. Poe M. P. Coughenour L. (2004). The Brief Assessment of Cognition in Schizophrenia: reliability, sensitivity, and comparison with a standard neurocognitive battery. *Schizophr. Res.* 68 283–297. 10.1016/j.schres.2003.09.011 15099610

[B18] Keles N. Caliskan M. Dogan B. Aksu F. Bulur S. Keles N. N. (2016). Is low serum klotho level associated with alterations in coronary flow reserve? *Echocardiography* 33 881–888. 10.1111/echo.13176 26791446

[B19] Kim H. J. Lee J. Chae D. W. Lee K. B. Sung S. A. Yoo T. H. (2019). Serum klotho is inversely associated with metabolic syndrome in chronic kidney disease: results from the KNOW-CKD study. *BMC Nephrol.* 20:119. 10.1186/s12882-019-1297-y PMC6446407 30943913

[B20] Kuro-O M. Matsumura Y. Aizawa H. Kawaguchi H. Suga T. Utsugi T. (1997). Mutation of the mouse klotho gene leads to a syndrome resembling ageing. *Nature* 390 45–51. 10.1038/36285 9363890

[B21] Kurosu H. Yamamoto M. Clark J. D. Pastor J. V. Nandi A. Gurnani P. (2005). Suppression of aging in mice by the hormone Klotho. *Science* 309 1829–1833. 10.1126/science.1112766 16123266 PMC2536606

[B22] Luo M. Zhou X. Ji H. Ma W. Liu G. Dai D. (2015). Population difference in the associations of KLOTH promoter methylation with mild cognitive impairment in xinjiang uygur and han populations. *PLoS One* 10:e0132156. 10.1371/journal.pone.0132156 26197428 PMC4509908

[B23] Lystad J. U. Falkum E. Haaland V. O. Bull H. Evensen S. Mcgurk S. R. (2017). Cognitive remediation and occupational outcome in schizophrenia spectrum disorders: a 2year follow-up study. *Schizophr. Res.* 185 122–129. 10.1016/j.schres.2016.12.020 28041917

[B24] Martin-Nunez E. Donate-Correa J. Ferri C. Lopez-Castillo A. Delgado-Molinos A. Hernandez-Carballo C. (2020). Association between serum levels of Klotho and inflammatory cytokines in cardiovascular disease: a case-control study. *Aging* 12 1952–1964. 10.18632/aging.102734 31986490 PMC7053623

[B25] McCleery A. Ventura J. Kern R. S. Subotnik K. L. Gretchen-Doorly D. Green M. F. (2014). Cognitive functioning in first-episode schizophrenia: MATRICS Consensus Cognitive Battery (MCCB) Profile of Impairment. *Schizophr. Res.* 157 33–39. 10.1016/j.schres.2014.04.039 24888526 PMC4112962

[B26] Medalia A. Saperstein A. M. (2013). Does cognitive remediation for schizophrenia improve functional outcomes? *Curr. Opin. Psychiatry* 26 151–157. 10.1097/yco.0b013e32835dcbd4 23318663

[B27] Morar B. Badcock J. C. Phillips M. Almeida O. P. Jablensky A. (2018). The longevity gene Klotho is differentially associated with cognition in subtypes of schizophrenia. *Schizophr. Res.* 193 348–353. 10.1016/j.schres.2017.06.054 28673754

[B28] Nagai T. Yamada K. Kim H. C. Kim Y. S. Noda Y. Imura A. (2003). Cognition impairment in the genetic model of aging klotho gene mutant mice: a role of oxidative stress. *FASEB J.* 17 50–52. 10.1096/fj.02-0448fje 12475907

[B29] Owen M. J. Sawa A. Mortensen P. B. (2016). Schizophrenia. *Lancet* 388 86–97. 26777917 10.1016/S0140-6736(15)01121-6 PMC4940219

[B30] Sastre C. Rubio-Navarro A. Buendia I. Gomez-Guerrero C. Blanco J. Mas S. (2013). Hyperlipidemia-associated renal damage decreases Klotho expression in kidneys from ApoE knockout mice. *PLoS One* 8:e83713. 10.1371/journal.pone.0083713 24386260 PMC3875485

[B31] Shin E. J. Chung Y. H. Le H. L. Jeong J. H. Dang D. K. Nam Y. (2014). Melatonin attenuates memory impairment induced by Klotho gene deficiency via interactive signaling between MT2 receptor, ERK, and Nrf2-related antioxidant potential. *Int. J. Neuropsychopharmacol.* 18:pyu105. 10.1093/ijnp/pyu105 PMC4438546 25550330

[B32] Shiozaki M. Yoshimura K. Shibata M. Koike M. Matsuura N. Uchiyama Y. (2008). Morphological and biochemical signs of age-related neurodegenerative changes in klotho mutant mice. *Neuroscience* 152 924–941. 10.1016/j.neuroscience.2008.01.032 18343589

[B33] Wang L. J. Lin P. Y. Lee Y. Huang Y. C. Wu C. C. Hsu S. T. (2018). Increased serum levels of cysteine in patients with schizophrenia: a potential marker of cognitive function preservation. *Schizophr. Res.* 192 391–397. 10.1016/j.schres.2017.03.041 28363347

[B34] Wu J. Q. Chen D. C. Tan Y. L. Xiu M. H. De Yang F. Soares J. C. (2016). Cognitive impairments in first-episode drug-naive and chronic medicated schizophrenia: MATRICS consensus cognitive battery in a Chinese Han population. *Psychiatry Res.* 238 196–202. 10.1016/j.psychres.2016.02.042 27086233

[B35] Xie T. Li Q. Luo X. Tian L. Wang Z. Tan S. (2019). Plasma total antioxidant status and cognitive impairments in first-episode drug-naive patients with schizophrenia. *Cogn. Neurodyn.* 13 357–365. 10.1007/s11571-019-09530-3 31354881 PMC6624222

[B36] Xiong J. W. Wei B. Li Y. K. Zhan J. Q. Jiang S. Z. Chen H. B. (2018). Decreased plasma levels of gasotransmitter hydrogen sulfide in patients with schizophrenia: correlation with psychopathology and cognition. *Psychopharmacology* 235 2267–2274. 10.1007/s00213-018-4923-7 29777287

[B37] Yamamoto M. Clark J. D. Pastor J. V. Gurnani P. Nandi A. Kurosu H. (2005). Regulation of oxidative stress by the anti-aging hormone klotho. *J. Biol. Chem.* 280 38029–38034. 10.1074/jbc.m509039200 16186101 PMC2515369

[B38] Yazici E. Mutu Pek T. Guzel D. Yazici A. B. Akcay Ciner O. Erol A. (2019). Klotho, vitamin D and homocysteine levels during acute episode and remission periods in schizophrenia patients. *Nord. J. Psychiatry* 73 178–184. 10.1080/08039488.2019.1582697 30896269

[B39] Yu Z. M. Zhao Y. Zhan J. Q. Luo T. Xiong J. W. Yu B. (2018). Treatment responses of cognitive function and plasma asymmetric dimethylarginine to atypical antipsychotic in patients with schizophrenia. *Front. Psychiatry* 9:733. 10.3389/fpsyt.2018.00733 30687138 PMC6335386

[B40] Zeng C. Y. Yang T. T. Zhou H. J. Zhao Y. Kuang X. Duan W. (2019). Lentiviral vector-mediated overexpression of Klotho in the brain improves Alzheimer’s disease-like pathology and cognitive deficits in mice. *Neurobiol. Aging* 78 18–28. 10.1016/j.neurobiolaging.2019.02.003 30851437

[B41] Zhang H. Wang Y. Hu Y. Zhu Y. Zhang T. Wang J. (2019). Meta-analysis of cognitive function in Chinese first-episode schizophrenia: MATRICS Consensus Cognitive Battery (MCCB) profile of impairment. *Gen. Psychiatr.* 32 e100043. 10.1136/gpsych-2018-100043 31423473 PMC6677937

[B42] Zhang Y. Fang X. Fan W. Tang W. Cai J. Song L. (2018). Brain-derived neurotrophic factor as a biomarker for cognitive recovery in acute schizophrenia: 12-week results from a prospective longitudinal study. *Psychopharmacology* 235 1191–1198. 10.1007/s00213-018-4835-6 29392373

[B43] Zhou H. J. Zeng C. Y. Yang T. T. Long F. Y. Kuang X. Du J. R. (2018). Lentivirus-mediated klotho up-regulation improves aging-related memory deficits and oxidative stress in senescence-accelerated mouse prone-8 mice. *Life Sci.* 200 56–62. 10.1016/j.lfs.2018.03.027 29544758

